# Plant Polysaccharides in Alzheimer’s Disease: From Phytochemistry to Microbiota-Gut–Brain Axis Mechanisms—Resolving the Pharmacokinetic-Pharmacodynamic Paradox

**DOI:** 10.3390/molecules31152622

**Published:** 2026-07-28

**Authors:** Jie Gao, Liheng Li, Qi Liu, Ning Zhang, Yan Li

**Affiliations:** 1 Heilongjiang University of Chinese Medicine, Harbin 150040, China; 2 Key Laboratory of Chinese Medicine Informatics of Heilongjiang Province, Harbin 150040, China

**Keywords:** traditional Chinese medicine (TCM) polysaccharides, Alzheimer’s disease (AD), short-chain-fatty acids (SCFAs), neuroinflammation, pharmacokinetic-pharmacodynamic (PK-PD) paradox

## Abstract

Alzheimer’s disease (AD) is a neurodegenerative disorder characterized by Aβ deposition, tau hyperphosphorylation, and neuroinflammation. No effective drugs can slow disease progression. Polysaccharides from traditional Chinese medicine (TCM) exhibit neuroprotective activities (e.g., antioxidant, anti-inflammatory) with good safety. However, their clinical application is limited by low oral bioavailability, poor blood–brain barrier (BBB) permeability, and a pharmacokinetic–pharmacodynamic paradox. The emerging role of the microbiota–gut–brain axis in AD offers a strategy to overcome this paradox. This review summarizes the structural features and classification of TCM polysaccharides (from plants, fungi, and roots/rhizomes) and highlights their anti-AD mechanisms via the gut–brain axis. Acting as prebiotics, these polysaccharides escape upper digestion and are fermented by gut microbiota into short-chain fatty acids (SCFAs) and other metabolites, which enter circulation, cross the BBB, and alleviate AD pathology through metabolic, immune, and neuronal pathways. Outcomes include reduced Aβ deposition and tau phosphorylation, suppressed neuroinflammation, restored synaptic function, and improved cognition. This review provides a theoretical framework for TCM polysaccharide intervention in AD via the gut–brain axis and a pharmacological basis for developing natural product-based AD therapies.

## 1. Introduction

Alzheimer’s disease (AD) is a progressive neurodegenerative disorder. As the disease progresses, cognitive capabilities gradually deteriorate, with memory impairment and diminished learning ability [1]. Amid the global increase in the elderly population, AD has emerged as a major and unignorable public-health concern. With over 50 million people worldwide affected by this condition, it imposes not only significant burdens on family caretakers but also substantial challenges on society as a whole [2,3]. Clinical features of AD are the accumulation of amyloid-β plaques and the presence of neurofibrillary tangles in the cerebral cortex—essentially abnormal clusters of hyperphosphorylated tau protein [4]. Recently conducted investigations have revealed the intricacies of the pathogenic processes underlying AD, which comprise a series of events—including neuroinflammation, redox-mediated oxidative damage, disruption of mitochondrial metabolism, impaired autophagy, endoplasmic reticulum protein misfolding, and others [5]. Although scientists have devoted considerable effort to studying these disease-related pathways, when it comes to daily clinical practice, we still lack drugs that can truly alter the course of the disease and offer what could be described as a “root-cause” cure.

The GBA acts as a multi-directional signaling hub connecting the enteric microbiota to the CNS via neural, immuno-endocrine, and metabolic-endocrine pathways [6,7]. In years past, a surge of studies confirmed this axis’s pivotal role in modulating AD pathogenesis, paving the way for novel future therapeutic development [8,9]. Notably, numerous clinical studies and animal experiments have revealed that the gut microbiota of AD patients is significantly disrupted—a condition known as dysbiosis. Messages communicated through the microbiota-gut–brain axis (MGBA) signaling pathway further influence the core aspects of this disorder, encompassing β-amyloid deposits, tau phosphorylation, and brain inflammation [10]. When viewed collectively, the findings provide a firm theoretical basis for the notion that regulating the gut microbiota could serve as a potential therapeutic approach for AD [11].

In TCM, herbs rich in polysaccharides have actually been used for a very long time. In ancient times, they were said to “tonify qi,” “enhance intelligence,” and “nourish the brain [12,13].” Current pharmacological research confirms that polysaccharides extracted from Chinese herbs improve cognitive deficits primarily through two mechanisms: first, by suppressing inflammatory responses in the brain, and second, by modulating the composition of the gut microbiota [14]. Interestingly, these polysaccharides are typically regarded as having a low gut absorption rate and difficulty traversing the BBB; nevertheless, their significant neuroprotective function in the central neuroaxis has been well-established [15]. This apparent contradiction is explained by the gut microbiota: through regulating microbial metabolites, polysaccharides modulate brain function [16,17]. Recent studies have also confirmed that the gut microbiota plays a key mediating role in regulating the gut–brain axis and influencing neurodegenerative diseases. This explains why naturally derived bioactive compounds can exert neuroprotective effects via this axis [18]. Overall, this line of reasoning not only provides a solid pharmacological basis for using polysaccharides from TCM to combat AD but also charts a new path for the modernization of TCM and the discovery of new drugs. This review, therefore, bridges phytochemical characterization of polysaccharides with their gut microbiota-driven molecular mechanisms, offering an integrated framework that resolves the long-standing PK–PD paradox and highlights therapeutic opportunities for AD. The literature covers the period from 2010 to 2026, with emphasis on studies of the structural features, microbiota-mediated metabolism, and neuroprotective mechanisms of TCM polysaccharides in AD models.

## 2. The Association and Core Mechanisms Between the Microbiota-Gut–Brain Axis and AD

The gut–brain axis is a bidirectional system connecting the gastrointestinal tract with the CNS through neural, immune, endocrine, and metabolic pathways, exerting profound influence on AD pathogenesis and progression [19]. From a neural perspective, gut microbiota transmits regulatory signals to the brain via the vagus nerve; microbial disruption suppresses GABA and BDNF synthesis, accelerating neurodegeneration [20,21,22,23]. At the immune interface, dysbiosis compromises intestinal barrier integrity, permitting bacterial components such as LPS to enter the bloodstream and instigate systemic inflammation [24]. These circulating mediators cross the compromised blood–brain barrier, hyperactivate microglia, and elicit neuroinflammatory responses within the CNS [25]. Metabolically, SCFAs derived from gut microbial fermentation participate in peripheral energy homeostasis and exert neuroprotective effects upon accessing the CNS [26,27]. Clinical evidence indicates that AD patients harbor fewer SCFA-producing bacteria, a deficiency that exacerbates neuroinflammation and correlates with cognitive decline [28,29]. Collectively, these pathways converge into a “leaky gut–inflammation–neuroinflammation” axis that fundamentally drives AD neuropathology.

The distinctive gut microbial composition in AD patients lends credence to this framework. 16S rRNA sequencing has documented dysbiosis in the AD gut microbiome, with reduced α-diversity and altered β-diversity [30,31]. This microbial imbalance correlates with AD pathology progression [32]. SCFA-producing commensals, including Faecalibacterium and Bifidobacterium, are diminished in AD patients, whereas pro-inflammatory Proteobacteria taxa are expanded [33]. Among individuals with cognitive impairment and cerebral β-amyloid deposition, Escherichia coli abundance is elevated, while Faecalibacterium populations are reduced [10]. AD severity parallels deterioration of gut microbial community structure [17]. This dysbiotic state reduces SCFA production, downregulates tight junction proteins, and increases intestinal permeability, facilitating systemic dissemination of LPS and other noxious metabolites that propagate neuroinflammation, Aβ aggregation, and tau hyperphosphorylation—a pathogenic cascade corroborated in animal models [9,24,34,35,36,37,38,39].

## 3. TCM Polysaccharides: Structural Characteristics and Regulation of Intestinal Flora Metabolism

### 3.1. Phytochemical Diversity of TCM Polysaccharides: Structural Characteristics and Classification

To fully understand the structure-activity relationship (SAR) and pharmacological effects of polysaccharides in TCM, it is essential to examine how their structural characteristics vary with source [40,41]. Based on biological origin, TCM polysaccharides fall into two major groups: plant-derived (including root, fruit, and rhizome subtypes) and fungal-derived polysaccharides (Table 1). The plant-derived group shows substantial structural diversity across subtypes, each with distinct molecular weight, monosaccharide composition, and glycosidic linkage profiles [42].

TCM polysaccharides exhibit molecular weights spanning from several thousand to millions of daltons; lower-MW fractions generally show better solubility and fermentation efficiency, whereas higher-MW counterparts display extended gastrointestinal retention. Their monosaccharide composition mainly comprises glucose, galactose, arabinose, rhamnose, mannose, xylose, and various uronic acids; polysaccharides rich in uronic acids resist enzymatic hydrolysis, thereby enhancing their availability for colonic fermentation. Glycosidic bonds occur as α- or β-types, with primary linkages at 1→2, 1→3, 1→4, and 1→6; α-linkages are more readily degraded by host enzymes, whereas β-linkages reach the colon intact for microbial metabolism. Collectively, these structural features govern microbial recognition, degradation, fermentation kinetics, SCFA profiles, and ultimately neuroprotective outcomes [43,44,45].

Among these features, several parameters critically influence bioactivity. Fungal polysaccharides, typified by Ganoderma lucidum polysaccharides (GLP), possess a β-(1→3)-D-glucan backbone with β-(1→6) side chains, forming a triple-helix conformation essential for immunomodulatory activity [46,47]. Branching degree modulates enzymatic accessibility; highly branched structures generally exhibit stronger activity [47]. Sulfate modification introduces negative charges that potentiate antioxidant capacity, whereas acetylation alters conformation and hydrophobicity, modulating interactions with gut microbiota and immune cells [43,45]. Collectively, these phytochemical parameters—molecular weight, monosaccharide composition, glycosidic linkage patterns, branching degree, and chemical modifications—govern microbial recognition and fermentation kinetics, thereby establishing a direct structural basis for MGBA-mediated neuroprotection. The structural features and neuroprotective mechanisms of representative polysaccharides are summarized in Table 1.

**Table 1:** Structural Characteristics and neuroprotective mechanisms of Key TCM Polysaccharides.

Name	Source Category (Specific Part)	Molecular Weight Range (kDa)	Major Monosaccharide Composition	Types of Glycosidic Bonds	Neuroprotective Mechanisms	References
Astragalus polysaccharides (APS)	Plant-derived(rhizome)	20–50	Glc, Ara, Gal	α/β type, 1→4/1→6 linkages	Modulates gut microbiota homeostasis by increasing beneficial bacteria (Lactobacillus/Bifidobacterium), suppresses TLR4/NF-κB signaling to reduce neuroinflammation, and enhances intestinal barrier integrity via SCFA production.	[48]
Lycium barbarum polysaccharides (LBP)	Plant-derived(fruit)	40–200	Ara, Gal, Glc, Rha	α-type, 1→3/1→4 linkages	Restores gut microbiota, promotes SCFA production and intestinal barrier protection; activates ERK/CREB/BDNF pathway to enhance synaptic plasticity; reduces Aβ deposition and tau hyperphosphorylation.	[49,50,51,52]
Ganoderma lucidum polysaccharides (GLP)	Fungal-derived(fruiting body)	10–50	Glc, Gal, Man	β-(1→3) main chain, β-(1→6) side chain, triple-helix conformation	Regulates gut microbiota by reducing F/B ratio and pro-inflammatory bacteria; promotes SCFA secretion to inhibit microglial activation and CNS inflammation.	[43,53]
Hericium erinaceus polysaccharides (HEP)	Fungal-derived(fruiting body)	~9000	Glc, Gal, Man	β-type, 1→3/1→4 linkages	Modulates gut microbiota fermentation to promote SCFA production, reduces peripheral inflammation, regulates immune factor secretion to alleviate CNS inflammation and improve cognitive function.	[54]
Polygala tenuifolia polysaccharides (PTP)	Plant-derived(rhizome)	~87	Ara, Rha, Gal, Glc	α/β-type, 1→2/1→4 linkages	Modulates gut microbiota by reducing pro-inflammatory bacteria, inhibits TLR4/NF-κB pathway to suppress neuroinflammation and Aβ aggregation/deposition.	[55,56,57]
Acorus tatarinowii polysaccharides (ATP)	Plant-derived (rhizome)	~87	Ara, Rha, Gal, Glc, Xyl, Man	Complex glycosidic bonds (α/β, 1→3/1→4/1→6)	Restores gut microbiota by promoting beneficial bacteria, inhibits TLR4/MyD88/NF-κB and PI3K/Akt pathways to reduce neuroinflammation and neuronal damage.	[58]

### 3.2. Mechanism Analysis of the Pharmacokinetic Paradox: From Polysaccharide Biotransformation to Short-Chain Fatty Acid Mediation

#### 3.2.1. The PK-PD Paradox of Orally Administered TCM Polysaccharides

Oral TCM polysaccharides present a pharmacokinetic-pharmacodynamic (PK-PD) paradox in anti-AD research: intact polysaccharide macromolecules exhibit negligible intestinal absorption and minimal blood–brain barrier (BBB) penetration, yet numerous rodent studies consistently demonstrate robust neuroprotection [59,60]. This discrepancy cannot be attributed to direct systemic actions of the parent compounds; instead, gut microbiota-mediated biotransformation constitutes the mechanistic foundation for this phenomenon.

The glycosidic bonds of herbal polysaccharides resist hydrolysis by host digestive enzymes in the stomach and small intestine. Consequently, most intact polysaccharides traverse the upper digestive tract without degradation and accumulate in the colon, where microbial density and diversity are highest [61,62]. Colonic microbiota, dominated by Firmicutes and Bacteroidetes, secrete carbohydrate-active enzymes (CAZymes) that recognize and cleave polysaccharide glycosidic linkages, progressively depolymerizing large molecules into fermentable oligosaccharides and monosaccharides [63,64].

**Figure 1:**
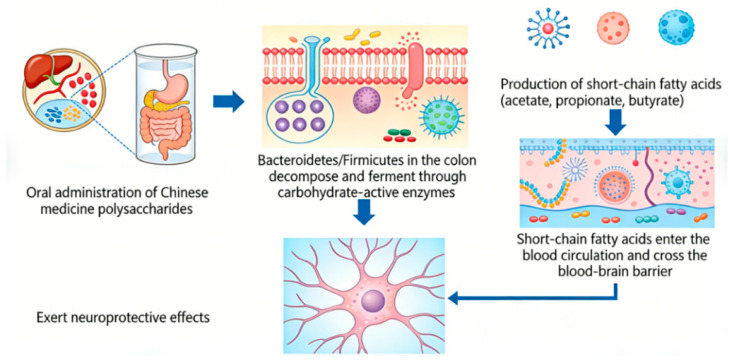
TCM polysaccharides reach the colon intact after oral administration. Gut microbes ferment these polysaccharides using CAZymes to produce SCFAs, which subsequently cross the BBB and deliver neuroprotective actions—thereby resolving the paradox of their low bioavailability yet notable oral efficacy.

Beyond short-chain fatty acids (SCFAs), microbial polysaccharide fermentation generates additional bioactive metabolites that participate in gut–brain axis signaling: Secondary bile acids cross the BBB and attenuate neuroinflammation via FXR and TGR5 signaling; Tryptophan-derived metabolites (indole-3-propionic acid, kynurenine) act as aryl hydrocarbon receptor ligands, modulating peripheral and central immunity; bacterial extracellular vesicles transport microbial molecules across the intestinal epithelium toward the CNS. Microbial neurotransmitters (GABA, dopamine, serotonin) are produced by gut bacteria and influence CNS function through vagal or systemic routes [22,23]. The entire process—from oral administration of intact polysaccharides to their colonic fermentation by gut microbiota and subsequent SCFA-mediated neuroprotection—is summarized schematically in Figure 1.

#### 3.2.2. SCFAs: The Principal Fermentation Metabolites Resolving the Paradox

Acetate, propionate, and butyrate constitute the primary end products of polysaccharide fermentation [6,26]. Unlike their high-molecular-weight precursors, SCFAs have molecular masses below 100 Da, conferring favorable pharmacokinetic attributes: rapid colonic absorption, efficient systemic distribution, and ready BBB permeation [65,66]. For instance, butyrate-producing bacteria such as Clostridium butyricum have been shown to enhance cognitive function in Alzheimer‘s disease models by modulating neuropathology and regulating acetic acid levels in the gut microbiota [67].

SCFA entry into the CNS is not governed by passive diffusion but is predominantly mediated by monocarboxylate transporters (MCTs), specifically MCT1 and MCT4, which are expressed on brain microvascular endothelial cells [68,69]. This carrier-mediated process displays regional heterogeneity, with greater transport efficiency in the hippocampus and hypothalamus relative to other cerebral regions [70]. SCFA brain access also exhibits dose dependence and is subject to extensive first-pass metabolism; a substantial portion of portal-derived SCFAs is rapidly extracted by the liver, thereby restricting their systemic bioavailability [71]. Collectively, these pharmacokinetic characteristics complicate the direct translation of peripheral SCFA concentrations into central neuroprotective efficacy and underscore the necessity of well-designed pharmacokinetic investigations to define precise dose–response relationships.

This biotransformation pathway resolves the PK-PD paradox: although intact polysaccharides do not reach the CNS, their fermentation products—SCFAs—cross the BBB and exert pleiotropic neuroprotective effects against AD pathology [17]. The origins, physiological functions, pharmacokinetic properties, and AD-related mechanisms of the three principal SCFAs are summarized in Table 2.

### 3.3. The Core Mechanism of TCM Polysaccharides Regulating the Gut–Brain Axis in the Intervention of AD

**Figure 2:**
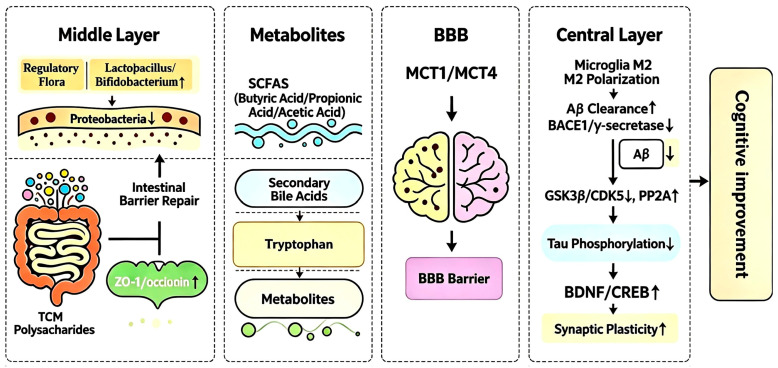
Illustrates that TCM polysaccharides, metabolized by gut microbiota to SCFAs, exert anti-AD effects via metabolic remodeling, immune modulation, and neuroprotection, targeting Aβ/tau pathology and improving cognitive function. Notation: Arrows indicate directional regulatory relationships; ↑ denotes elevation, ↓ denotes suppression. Distinct colored backgrounds separate four functional segments of the gut–brain communication cascade for clearer visualization and carry no specific biological significance.

The therapeutic advantages of TCM polysaccharides in AD do not stem from direct effects on the CNS, but rather rely on the gut microbiota as a key intermediary. This interaction mobilizes the MGBA through several pathways, thereby helping us gain a more comprehensive understanding of the pathological progression of AD. The primary mechanisms involved—namely, metabolic remodeling, immune regulation, and neuroprotection—are summarized in Figure 2. These components do not operate in isolation but are interconnected and mutually reinforcing, ultimately forming an interlinked regulatory chain. To illustrate, the pathway can be summarized as “polysaccharides → gut microbiota → metabolites → MGBA → neuroprotection” [12,71].

### 3.4. Metabolic Remodeling: Polysaccharide-Gut Microbiota-SCFA Interaction Axis

As discussed above, the structural diversity of TCM polysaccharides determines their utilization by gut microbiota and subsequent SCFA production. The distinct metabolic profiles of different polysaccharide types are summarized in Table 3.

Once SCFA levels in the gut decline, intestinal barrier integrity becomes compromised, which in turn triggers neuroinflammation [10,31]. TCM polysaccharides, by serving as fermentable substrates, initiate a beneficial cycle of nutrient supply, microbial proliferation, and metabolite production. This process not only restores intestinal SCFA concentrations but also modulates bidirectional signaling along the gut–brain axis [14,17].

SCFAs, functioning as principal metabolic messengers of the MGBA, coordinate a regulatory axis that connects intestinal metabolism with systemic signaling and central neuroprotection. Through engagement with G protein-coupled receptors, these metabolites modulate microglial polarization and suppress the release of pro-inflammatory mediators [27,67]. Concurrently, AMPK-mediated upregulation of tight junction proteins reinforces intestinal epithelial integrity, thereby limiting the translocation of endotoxins and inflammatory stimuli into the circulation [24,34]. At the level of the central nervous system, SCFAs act directly upon the BDNF/CREB transcriptional cascade and histone deacetylase activity, leading to enhanced synaptic plasticity and attenuated APP processing as well as tau hyperphosphorylation [28,29,72].

### 3.5. Immune Regulation: Inhibiting Neuroinflammation and Reshaping the Immune Microenvironment

Neuroinflammation permeates the entire pathological course of AD and is a core factor driving its progression. The “leaky gut–inflammation” cascade triggered by gut microbiota dysbiosis is a key mechanism underlying central neuroinflammation [8,9]. TCM polysaccharides primarily target the gut microbiota, synergistically reshaping the immune landscape at both the peripheral and central levels. This disrupts a vicious cycle—“gut microbiota dysbiosis → peripheral inflammation → central inflammation”—thereby enabling a systemic immunotherapeutic strategy for AD [13,73].

Polysaccharides derived from TCM interact with the gut microbiota and its metabolites to first regulate peripheral immune balance [20]. At the same time, they can directly act on immune cells in the central nervous system, exerting a neuroprotective effect [48]. In the peripheral system, these polysaccharides maintain the balance of immune cell subsets, suppress pro-inflammatory signals along the TLR4/NF-κB pathway, and reduce the production of pro-inflammatory mediators, thereby preventing peripheral inflammation from spreading to the CNS [72]. In the CNS, they alter the polarization state of microglia, regulate astrocyte activation, reduce neuroinflammation, and simultaneously enhance the secretion of neurotrophic factors to aid neuronal repair. They exert layer-by-layer regulation along the axis of “gut microbiota → peripheral immunity → central immunity,” ultimately providing immune-level support for neuronal protection [12,58,60].

### 3.6. Neuroprotection: The Integrated Effect of Targeting Core Pathology and Repairing Synaptic Function

Neuroprotection represents the ultimate outcome of TCM polysaccharide effects exerted via the gut–brain axis. It is achieved by integrating metabolic reprogramming and immune regulation at the neuronal level [74,75]. Through metabolic reprogramming, these polysaccharides deliver key signaling molecules such as short-chain fatty acids; through immune modulation, they reduce neuroinflammation and, in the process, improve the environment in which neurons thrive. Together, these two mechanisms directly target the core pathological features of AD. Leveraging the regulatory functions of short-chain fatty acids, the entire process coordinates a series of interlinked chain reactions [76,77].

Polysaccharides derived from TCM act through the short-chain fatty acid pathway to simultaneously influence both the production and clearance of Aβ, thereby achieving bidirectional regulation of Aβ metabolism [78,79,80]. On the one hand, they limit Aβ production by inhibiting secretases such as BACE1 and γ-secretase; on the other hand, they aid in Aβ clearance by promoting the polarization of microglia toward the M2 phenotype and enhancing transport functions at the blood–brain barrier. Together, these mechanisms ultimately restore Aβ levels to equilibrium in the brain [81,82,83]. At the same time, these polysaccharides finely regulate the kinase–phosphatase system via short-chain fatty acids, maintaining the phosphorylation homeostasis of tau protein and thereby correcting dysregulation of enzymes such as GSK3β and CDK5 [84,85]. It is worth noting that PP2A is a major serine/threonine phosphatase that negatively regulates phosphorylation-driving kinases such as GSK3β and governs cellular dephosphorylation pathways [86]. By maintaining the phosphorylation balance of tau protein, this regulatory network limits the formation of neurofibrillary tangles, thereby preserving neuronal integrity [87].

By effectively regulating pathological protein aggregation, polysaccharides derived from TCM can help restore synaptic function and improve the environment in which neurons thrive—both of which are crucial for reversing cognitive decline in AD patients [66]. These structurally complex polysaccharides use short-chain fatty acids as signaling messengers to actively repair synaptic damage, which is precisely a major cause of cognitive deficits in AD [76]. By activating the BDNF/CREB pathway, they increase the production of synaptic-related proteins and promote synaptic plasticity [48,73]. This stepwise regulation—from “clearing pathological proteins” to “restoring synaptic function”—not only mitigates AD-related neuropathological damage but also provides comprehensive protection for neuronal structure and function. The specific targets, underlying mechanisms, and representative polysaccharides for each regulatory step are summarized in Table 4.

### 3.7. Research Progress on the Intervention of AD by Polysaccharides from Different Sources of TCM Through the MGBA

Building on the structural classification above, polysaccharides from different sources exhibit significant differences in substrate specificity toward the gut microbiota and their resulting metabolic profiles [90]. For the purposes of mechanistic discussion via the MGBA, these are consolidated into two main categories—plant-derived polysaccharides (encompassing root, fruit, and rhizome subtypes) and fungal-derived polysaccharides—as their functional convergence in anti-AD mechanisms justifies this grouping.

#### 3.7.1. Polysaccharides from Plant-Based TCM

Plant-derived polysaccharides play a significant role in AD intervention. APS exert neuroprotective effects through multiple mechanisms, including promoting SCFA-producing microbiota, restoring intestinal barrier integrity, and modulating the MAPK/NF-κB pathway to alleviate neuroinflammation [77,91]. LBPs enhance synaptic plasticity via neurotransmitter balance within the MGBA [49,50,51,52]. GSPM activate AMPK/Sirt1 through gut microbiota modulation [92], while Pueraria lobata polysaccharides inhibit LPS/TLR4-driven neuroinflammation by repairing the intestinal barrier [75].

Other root and rhizome polysaccharides also act through the MGBA: ATP blocks LPS/TLR4/MyD88 signaling to suppress neuroinflammation [58]; PF downregulates CDK5/GSK-3β to reduce tau phosphorylation [93]; Codonopsis pilosula polysaccharides reduce BACE1 activity [94]; PSP enhances microglial Aβ phagocytosis [76]; PTPS activates ERK to reduce Aβ deposition [57]; ACP reshapes gut microbiota to improve brain pathology [95]; PEP regulates autophagy and oxidative stress [96]; RHP protects hippocampal mitochondria and promotes autophagy [97]; GEP increases neuronal numbers [98]; EOP and CDPS enrich beneficial microbiota and reduce cerebral Aβ levels [15,16,99]. See Table 5 for a comprehensive summary.

#### 3.7.2. Polysaccharides of TCM from Fungal Sources

Fungal polysaccharides have a unique triple-helix structure, and modification can enhance their bioactivity. Modified Ganoderma lucidum polysaccharides (GLP) promote fermentation, increase SCFA-producing microbiota, and improve cognition [12,53]. Hericium erinaceus polysaccharides (HEP) activate Nrf2 and modulate vagus nerve–MGBA signaling [54]. Flammulina velutipes polysaccharides regulate gut microbiota, strengthen the intestinal barrier, and suppress neuroinflammation [100].

Other fungal polysaccharides also act via the MGBA: Poria cocos polysaccharides (PCP) inhibit TLR4/NF-κB to alleviate neuroinflammation [14]; Sparassis crispa polysaccharides enhance GABA synthesis and improve cognition [88]. See Table 5 for details.

Among the polysaccharides examined in this review, several display promising neuroprotective efficacy in preclinical AD models. APS, LBP, and GLP consistently reduce Aβ deposition, suppress neuroinflammatory responses, and improve cognitive performance in APP/PS1 and SAMP8 transgenic mice, with APS additionally showing favorable tolerability and no overt toxicity [48,50,101,102,103]. HEP and PTP exert marked effects on synaptic plasticity and tau phosphorylation [56]. Regarding safety, most TCM polysaccharides have demonstrated acceptable tolerability in preclinical studies, with no significant adverse events reported at therapeutic doses [15,87]. Nonetheless, comprehensive toxicological evaluations—particularly long-term safety monitoring and immunogenicity assessments—remain inadequate. The intrinsic heterogeneity of polysaccharide preparations, together with the absence of standardized administration protocols, further complicates cross-study comparisons of both efficacy and safety. Future work should prioritize rigorous characterization and systematic toxicological profiling to facilitate clinical translation.

## 4. Discussion

### 4.1. Core Mechanism Resolving the PK-PD Paradox

The resolution of the PK-PD paradox lies in the recognition that TCM polysaccharides function not as conventional CNS-targeting agents, but rather as prodrug-like substrates that are metabolically transformed by the gut microbiota. Their fermentation yields bioactive small-molecule metabolites that gain access to the brain through systemic circulation, thereby establishing a gut-to-brain signaling axis that circumvents the requirement for direct absorption of the intact polysaccharides. This conceptual framework integrates phytochemical properties with in vivo pharmacological outcomes and positions TCM polysaccharides as a distinctive category of gut-oriented therapeutic agents for AD. This reconceptualization of the PK-PD relationship carries direct implications for clinical translation. Rather than relying solely on plasma concentrations of parent polysaccharides, therapeutic efficacy should be evaluated through integrated pharmacokinetic-pharmacodynamic models that incorporate gut microbial metabolism as a key variable. The identification of microbial metabolites—particularly SCFAs and other bioactive mediators—as functional readouts offers a rational basis for developing surrogate biomarkers for clinical trials [104]. Furthermore, the substantial inter-individual variability in gut microbial composition underscores the need for microbiome stratification to identify patient subgroups most likely to benefit from specific polysaccharide interventions [105]. These strategies collectively transform the PK-PD paradox from a translational barrier into a framework for precision therapeutics.

### 4.2. Controversies and Unresolved Issues

The structure-activity relationship (SAR) of TCM polysaccharides remains inadequately characterized, constituting a critical knowledge gap in the field. Accumulating evidence indicates that multiple structural determinants govern bioactivity: (i) molecular weight—lower-MW fractions generally afford enhanced solubility and fermentability, whereas higher-MW counterparts exhibit extended gastrointestinal retention; (ii) glycosidic linkage architecture—β-(1→3)-backboned polymers with β-(1→6)-branched side chains, characteristic of fungal polysaccharides, correlate with potent immunomodulatory properties, whereas α-linked polysaccharides display comparatively greater antioxidant potential; and (iii) uronic acid composition—polysaccharides enriched in galacturonic acid demonstrate augmented prebiotic efficacy. Nevertheless, these observed associations remain predominantly phenomenological. Several substantial obstacles impede further progress: the intrinsic heterogeneity of polysaccharide isolates confounds attribution of discrete bioactivities to well-defined structural moieties; the conformational equilibria (triple-helical versus random-coil states) and their influence on host microbial recognition remain largely unexplored; and the structure-dependent catabolic trajectories—namely, the manner in which specific glycosidic linkages dictate degradation kinetics and resultant SCFA profiles—have yet to be systematically elucidated. Resolution of these issues will necessitate integrative strategies incorporating controlled chemical derivatization, high-resolution NMR and mass spectrometric analysis, in vitro anaerobic fermentation platforms, and multi-dimensional omics technologies toward the establishment of robust, predictive SAR frameworks.

### 4.3. Current Research Gaps

That said, there are still several research gaps waiting to be filled. First, there has been a lack of systematic research on the exact relationship between structure-activity relationships and metabolite profiles, a gap that must be addressed through omics approaches. Second, current research is somewhat biased—it tends to focus solely on individual polysaccharide components while overlooking the potential synergistic effects among multiple polysaccharides in TCM formulas. Third, our understanding of gut–brain signaling pathways remains incomplete. For instance, we still do not fully understand where short-chain fatty acid receptors exert their effects or their specific binding sites. Fourth, clinical evidence is severely lacking; most conclusions are derived solely from rodent models. Fifth, there is another issue that is easily overlooked: each person’s gut microbiome composition is inherently unique, yet current intervention strategies fail to account for this, let alone enable personalized or stratified treatment.

### 4.4. Future Directions

To advance this field, there are several strategies worth exploring. First, we need to leverage machine learning to integrate multi-omics data and build models capable of predicting the relationship between polysaccharide structure and biological function. Second, we should develop targeted delivery systems—such as nanoparticles, hydrogels, and enteric-coated formulations—to protect the polysaccharides and prevent them from being degraded before they reach the colon. Third, we should utilize network pharmacology and systems biology methods to assess whether there are synergistic effects among the multiple components in TCM formulas. Fourth, we need to carefully design randomized controlled trials that not only measure cognitive function but also take into account gut microbiota profiles, short-chain fatty acid quantification, and microbial stratification within the population. Fifth, we should consider combining TCM polysaccharides with existing AD drugs to simultaneously target multiple pathological pathways. Sixth, the processes for extraction, purification, and characterization must be standardized; otherwise, clinical translation and regulatory approval will be difficult to advance.

### 4.5. Clinical Evidence and Translational Challenges

Despite the abundance of preclinical evidence supporting TCM polysaccharides against AD, human data remain remarkably limited. To date, no large-scale, placebo-controlled randomized trials have evaluated purified polysaccharides as standalone AD interventions. Available human evidence derives primarily from small-scale observational studies, open-label trials, and safety assessments of polysaccharide-containing herbal extracts rather than isolated fractions.

Among the few clinical studies conducted, some have examined the safety and tolerability of polysaccharide-rich TCM preparations. An open-label study of Cistanche tubulosa glycoside capsules (Memoregain^®^) in moderate AD patients reported favorable tolerability over 48 weeks, with mild gastrointestinal events as the most common adverse effects [1]. A randomized, double-blinded, placebo-controlled trial of Ganoderma lucidum-derived β-1,3;1,6-D-glucan in healthy adults demonstrated significant immunomodulatory effects on T-lymphocytes and natural killer cells, with no adverse effects on liver or kidney function over 12 weeks [106]. However, these studies were not powered to detect cognitive improvement, and their open-label or healthy-population designs preclude definitive efficacy conclusions in AD patients.

Human pharmacokinetic data are even scarcer. The low oral bioavailability of intact polysaccharides, together with a lack of validated biomarkers for their microbiota-derived metabolites, poses substantial challenges for trial design. Most pharmacokinetic studies have measured circulating SCFAs or other microbial metabolites after dietary fiber or polysaccharide intake, rather than tracking the parent compounds themselves [65]. This indirect approach reflects the PK-PD paradigm but complicates dose–response and exposure-effect evaluations.

Inter-individual variability in gut microbial composition presents another translational hurdle. Nearly all preclinical studies summarized in this review used inbred rodent strains under controlled conditions, which minimize microbiome diversity but fail to mirror heterogeneous human populations. The abundance of SCFA-producing bacteria and saccharolytic enzymes varies considerably across individuals, as demonstrated by population-level microbiome studies showing that host location and geography strongly associate with microbiota variations [107]. This suggests that therapeutic responses may differ markedly between subjects, underscoring the necessity of baseline microbiome profiling for participant stratification in future trials.

Regulatory challenges further impede clinical translation. The inherent heterogeneity of polysaccharide preparations, compounded by a lack of standardized extraction and characterization protocols, undermines reproducibility and cross-study comparability. Unlike small-molecule drugs with defined chemical structures, polysaccharides are complex, polydisperse macromolecules whose bioactivity depends on multiple interdependent structural parameters [40]. Establishing quality control standards—including batch-to-batch consistency, molecular weight distribution, and monosaccharide composition—is a prerequisite for regulatory approval.

In summary, the preclinical foundation for TCM polysaccharides in AD is robust, but the clinical evidence base remains nascent. Rigorous randomized controlled trials with adequate sample sizes, validated cognitive endpoints, microbiome stratification, and standardized preparations are urgently needed. Only through such investigations can the therapeutic promise of TCM polysaccharides be adequately evaluated and translated into patient benefit. To translate the PK-PD paradox into actionable clinical strategies, future research should prioritize three interconnected pathways: (i) establishing robust pharmacokinetic-pharmacodynamic models that incorporate gut microbial metabolism as a key variable, rather than relying solely on plasma concentrations of parent compounds [108]; (ii) developing validated biomarkers of polysaccharide fermentation (e.g., SCFA profiles, specific microbial taxa abundance) as surrogate endpoints for clinical trials; (iii) implementing microbiome stratification to identify patient subgroups most likely to benefit from specific polysaccharide interventions [109,110]. These approaches would transform the apparent PK-PD paradox from a translational barrier into a framework for precision therapeutics.

### 4.6. Personalized Medicine and Microbiome Stratification

The inherent individuality of the human gut microbiome represents one of the most significant challenges—and opportunities—for the clinical translation of TCM polysaccharide-based therapies. Given that polysaccharide fermentation profiles are largely determined by the composition and functional capacity of an individual’s gut microbial ecosystem, therapeutic responses to a given polysaccharide may vary considerably across populations. This variability manifests clinically as “responder” versus “non-responder” phenotypes, a phenomenon well documented in prebiotic intervention studies [111]. Individuals with high baseline abundance of SCFA-producing genera (e.g., Faecalibacterium, Roseburia, Bifidobacterium) may be more likely to benefit from polysaccharide supplementation than those with dysbiotic profiles characterized by reduced saccharolytic capacity [17,31].

Microbiome stratification—the classification of individuals according to their gut microbial enterotypes or functional gene clusters—offers a rational basis for patient selection and treatment allocation [112,113]. The integration of machine learning and artificial intelligence into this framework holds considerable promise; predictive algorithms trained on multi-omics datasets (metagenomic, metabolomic, and clinical parameters) could identify baseline microbial features that predict therapeutic outcomes, thereby enabling rational patient stratification [114]. Realizing the full potential of personalized microbiome-targeted therapies will require large-scale, well-phenotyped clinical cohorts and the development of validated predictive biomarkers.

### 4.7. Significance and Translational Implications

The findings synthesized in this review carry several important implications for both fundamental understanding and clinical translation. First, the resolution of the PK-PD paradox establishes a new conceptual framework for natural product pharmacology: rather than requiring direct CNS penetration, therapeutic efficacy can be achieved through gut microbial biotransformation [64], expanding the scope of bioactive compounds traditionally considered “undruggable” due to poor bioavailability. Second, the identification of multiple microbial mediators—SCFAs, secondary bile acids, tryptophan metabolites, neurotransmitters, and extracellular vesicles—suggests that TCM polysaccharides exert pleiotropic effects through convergent pathways [115,116], offering a mechanistic rationale for their observed multi-target actions in AD models. Third, the recognition of inter-individual variability in gut microbial composition underscores the necessity of personalized approaches, moving beyond the “one-size-fits-all” paradigm toward microbiome-guided patient stratification [117,118]. From a clinical perspective, these insights provide a pharmacological basis for developing standardized polysaccharide preparations as adjunctive or preventive therapies for AD, particularly in prodromal or early-stage populations where microbiome modulation may offer disease-modifying potential.

## 5. Conclusions

In summary, resolution of the PK–PD paradox positions TCM polysaccharides as a distinct class of natural compounds that act via the gut microbiota to generate neuroprotective metabolites, rather than by directly targeting CNS molecules. Although the existing evidence is strong, there is a need to progress from observational accounts to detailed mechanistic analysis, thorough clinical confirmation, and personalized application. By utilizing multi-omics tools, creative administration techniques, and personalized medicine, TCM polysaccharides that target the MGBA could become a revolutionary approach for preventing and treating AD.

## Figures and Tables

**Table 2:** Sources, physiological functions, pharmacokinetic characteristics, and anti-Alzheimer‘s disease mechanisms of three major short-chain fatty acids.

SCFA Type	Primary Source	Physiological Functions	Pharmacokinetic Advantages	Potential Effects on AD	References
Butyrate	Fermentation by Firmicutes	Major energy fuel for colonocytes; HDAC inhibitor	Promotes expression of tight junction proteins (ZO-1, occludin, claudin-1)	Enhances intestinal barrier integrity, reduces LPS translocation, and alleviates neuroinflammation.	[6,66,67]
Propionate	Fermentation by Bacteroidetes	Substrate for hepatic gluconeogenesis; exerts anti-inflammatory actions	Enters systemic circulation; preserves neuronal mitochondrial homeostasis via GPR41 signaling	Attenuates peripheral inflammation and confers CNS protection.	[27,29]
Acetate	Fermentation by most gut microbiota	Substrate for lipogenesis; functions as a signaling molecule	Crosses the BBB; directly accesses the CNS	Modulates microglial activity to suppress neuroinflammation.	[20,28]

**Table 3:** Metabolic characteristics of gut microbiota and differential SCFA generation from structurally diverse TCM polysaccharides.

Polysaccharide Type	Core Structural Features	Dominant Metabolizing Microbiota	Major SCFA Products	SCFA Production Efficiency Advantage	References
Fungal polysaccharides	β-(1→3)-D-glucan backbone with β-(1→6) branches, triple-helix	*Lactobacillus*, *Bacteroides*, *Bifidobacterium*	Total SCFAs significantly increased (including butyrate)	SCFA yield increased by >50% after 48 h of fermentation	[43,46]
Plant polysaccharides	Structurally diverse (e.g., Poria cocos polysaccharide as β-glucan)	Increased gut microbiota diversity; enrichment of SCFA-producing genera	Propionate, butyrate, acetate, etc.	Structure dictates metabolic efficiency; fermentation rates vary substantially by polysaccharide category.	[14,17,46]

**Table 4:** Multi-target mechanism of TCM polysaccharides in regulating the Core pathology of AD.

Regulatory Dimension	Target	Regulation Direction	Core Mechanism	References
Aβ metabolism regulation	BACE1, γ-secretase	Inhibition	Maintains gut microbiota homeostasis, which reduces Aβ burden and improves cognitive function.	[77]
	Microglia (M2 polarization)	Enhancement	SCFAs promote M2 polarization of microglia, enhancing Aβ clearance.	[81,82]
	LRP1, P-gp	Upregulation	Gut microbiota homeostasis reduces neuroinflammation and alleviates AD pathology.	[43,53,83]
	Aβ fibrillization/oligomerization	Inhibition	Suppresses TLR4/MyD88/NF-κB signaling to reduce neuroinflammation and Aβ neurotoxicity.	[55,57]
Tau phosphorylation regulation	GSK3β, CDK5	Inhibition	Inhibits GSK3β and CDK5 activity, reducing tau phosphorylation at Ser396/Thr231.	[86]
	PP2A	Activation	Activates PP2A to maintain tau phosphorylation balance and reduce aberrant phosphorylation at Thr231.	[58,87]
Synaptic function repair	Synaptic structure	Protection	Prevents synaptic loss and maintains synaptic structure and functional integrity.	[76]
	Cholinergic system	Improvement	Increases ChAT activity and decreases AChE activity, reducing ACh degradation and enhancing cholinergic signaling.	[88]
	GABA level	Enhancement	Modulates gut microbiota to promote GABA synthesis, elevating central GABA levels and maintaining excitation-inhibition balance.	[88]
	Overall mechanism	Multi-pathway protection	Reduces oxidative stress to promote neuronal survival; regulates metabolism to enhance intestinal barrier function.	[89]

**Table 5:** Summary of the anti-AD mechanism of TCM polysaccharides through the gut MGBA.

Polysaccharide Name (Abbreviation)	Source Category	Animal/Cell Models	Core Mechanisms via Gut Microbiota-Brain Axis in AD	References
*Astragalus polysaccharides (APS)*	Plant-derived	APP/PS1 mice, LPS-challenged mice, PC12 cells	Promotes SCFA-producing bacteria, enhances intestinal barrier integrity, suppresses neuroinflammation, reduces Aβ/tau pathology, and improves cognitive performance.	[77,91]
*Lycium barbarum polysaccharides (LBP)*	Plant-derived	Aβ_1–42_-injected mice, SAMP8 mice, SH-SY5Y cells	Increases propionate synthesis, enhances gut–brain neurotransmitter balance, promotes synaptic plasticity, and ameliorates Aβ1–42-induced cognitive impairment.	[49,50,51,52]
*Ginseng–Polygonum multiflorum formula (GSPM)*	Plant-derived	D-galactose-accelerated aging mice, APP/PS1 mice	Modulates gut microbiota composition, restores F/B ratio, enriches Lactobacillus, activates AMPK/Sirt1 pathway, and improves cognitive function.	[92]
*Pueraria lobata polysaccharides*	Plant-derived	LPS-challenged mice, APP/PS1 mice, cerebral ischemia model	Regulates gut microbiota, elevates SCFA levels, upregulates ZO-1/occludin, repairs intestinal barrier, suppresses LPS/TLR4 pathway, and alleviates neuroinflammation.	[75]
*Pseudostellaria heterophylla polysaccharides (PF)*	Plant-derived	Aβ_1–42_-treated zebrafish, SH-SY5Y cells	Downregulates CDK5/GSK-3β, reduces tau phosphorylation (Ser396/Thr231), and attenuates Aβ1–42-induced cognitive impairment.	[93]
*Ganoderma lucidum polysaccharides (GLP, sulfated modification)*	Fungal-derived	SAMP8 mice, aged mice, Aβ-exposed cells	Enhances gut microbiota fermentation efficiency, increases SCFA-producing bacteria, and improves learning and memory function.	[12,53]
*Hericium erinaceus polysaccharides (HEP)*	Fungal-derived	Scopolamine-treated mice, PC12 cells	Degradation products together with SCFAs activate the Nrf2 pathway, reduce oxidative stress, and modulate vagus nerve–GBA signaling.	[54]
*Flammulina velutipes polysaccharides*	Fungal-derived	Scopolamine-treated mice, RAW264.7 cells	Modulates gut microbiota (Lactobacillus/Bifidobacterium), repairs intestinal barrier, lowers TNF-α/IL-6, and suppresses neuroinflammation.	[100]
*Poria cocos polysaccharides (PCP)*	Fungal-derived	APP/PS1 mice, BV2 microglia	Reshapes gut microbiota, increases SCFA-producing bacteria, upregulates tight junction proteins, inhibits TLR4/NF-κB, and alleviates neuroinflammation.	[14]
*Sparassis crispa polysaccharides*	Fungal-derived	AD model mice, BV2 microglia	Regulates gut microbiota, enhances GABA synthesis, elevates central GABA levels, balances excitation–inhibition, and improves cognitive function.	[88]
*Acorus tatarinowii polysaccharides (ATP)*	Plant-derived	LPS-challenged mice, BV2 cells	Reduces Proteobacteria abundance, blocks LPS/TLR4/MyD88 pathway, repairs intestinal barrier, and suppresses central neuroinflammation.	[58]
*Asparagus cochinchinensis polysaccharides (ACP)*	Plant-derived	APP/PS1 mice, SAMP8 mice	Regulates gut microbiota composition, ameliorates brain pathology, and improves cognitive function.	[95]
*Phyllanthus emblica polysaccharides (PEP)*	Plant-derived	Scopolamine-treated mice, SAMP8 mice	Regulates autophagy-related proteins, reduces inflammation and oxidative stress, and reshapes gut microbiota.	[96]
*Hedysarum polysaccharides (RHP)*	Plant-derived	SAMP8 mice, primary hippocampal neurons	Enhances intestinal barrier function, protects hippocampal mitochondria, promotes neuronal autophagy, modulates GBA, and improves cognition.	[97]
*Polygonatum sibiricum polysaccharides (PSP)*	Plant-derived	APP/PS1 mice, primary neurons	Reshapes gut microbiota, enhances microglial Aβ phagocytosis, and reduces synaptic loss.	[76]
*Polygala tenuifolia polysaccharides (PTPS)*	Plant-derived	APP/PS1 mice, SH-SY5Y cells	Activates ERK pathway, reduces Aβ deposition, and enhances synaptic plasticity.	[56]
*Gastrodia elata homogeneous polysaccharides (GEP)*	Plant-derived	Aβ-exposed mice, PC12 cells	Reduces Aβ deposition, increases neuronal numbers, and improves cognitive function.	[98]
*Codonopsis pilosula polysaccharides*	Plant-derived	APP/PS1 mice, HT22 cells	Suppresses BACE1 activity, reduces Aβ production, maintains gut microbiota balance, and alleviates cerebral inflammation.	[94]
*Eucommia ulmoides polysaccharides (EOP)*	Plant-derived	Aβ_25–35_-injected mice, SAMP8 mice	Reshapes gut microbiota, enriches Akkermansia, regulates SCFA metabolism, and suppresses neuroinflammation.	[16]
*Cistanche tubulosa* polysaccharides (CDPS)	Plant-derived	APP/PS1 mice	Enriches Akkermansia and other beneficial bacteria, regulates fatty acid metabolism, and reduces cerebral Aβ levels.	[99]

## Data Availability

No new data were created or analyzed in this study. Data sharing is not applicable to this article.
